# Evaluating Adverse Outcomes After Myringotomy or Tube Placement in Head and Neck Cancer

**DOI:** 10.1002/ohn.1186

**Published:** 2025-03-19

**Authors:** Krithika Kuppusamy, Carly Y. Yang, Kevin Wong, Douglas C. Bigelow, Michael J. Ruckenstein, Steven J. Eliades, Jason A. Brant, Tiffany Hwa

**Affiliations:** ^1^ Department of Otolaryngology–Head and Neck Surgery University of Pennsylvania Health System Philadelphia Pennsylvania USA; ^2^ Department of Head and Neck Surgery & Communication Sciences Duke University Health System Durham North Carolina USA; ^3^ Department of Otolaryngology–Head and Neck Surgery University of Wisconsin Madison Wisconsin USA

**Keywords:** head and neck cancer, middle ear effusion, myringotomy, OME, tympanostomy, head and neck radiation

## Abstract

**Objective:**

Evaluate rates of adverse outcomes among patients with a history of head and neck cancer undergoing myringotomy with or without tube placement for middle ear effusion.

**Study Design:**

Retrospective chart review.

**Setting:**

Academic medical center.

**Methods:**

Retrospective chart review was performed on patients undergoing myringotomy with or without tube placement for middle ear effusion between 2018 and 2022. Data reviewed included demographics, cancer history, audiometry, and clinical course.

**Results:**

In total, 578 patients (736 ears) had a mean follow‐up of 36.6 months: 84 (14.53%) were in the cancer cohort. On average, cancer patients were older (62.6 vs 59.3 years, *P* < .05) but had similar rates of overall adverse outcomes (44.05% vs 44.13%, *P* = 1.0). Rates of persistent perforation were higher among cancer patients (14.29% vs 2.43%, *P* < .001); there was no significant difference in rates of recurrent effusion (5.95% vs 4.66%; *P* = .81). Postpropensity score matching, perforation rates reached statistical significance (14.29% vs 1.22%, *P* < .01). There was no difference in rate of adverse events for overall events (44.05% vs 47.56%, *P* = .77) or recurrent effusion (5.95% vs 1.22%, *P* = .22).

**Conclusion:**

Patients with a history of head and neck cancer or radiation have a three‐to‐five‐fold risk of persistent tympanic membrane perforation after myringotomy with or without tube placement and a higher rate of recurrent effusion that is not significant. In multivariate analysis, perforation risk was revealed to be multifactorial.

Middle ear effusion (MEE), also known as otitis media with effusion (OME), is the accumulation of fluid within the middle ear cavity without signs or symptoms of acute ear infection. The diagnosis is made based on one or more of the following criteria: a reduction in tympanic membrane (TM) mobility observed through pneumatic otoscopy or tympanometry, the presence of an opaque TM, or a visible air‐fluid interface behind the TM detected on otoscopy.^1^ Studies indicate that adults with certain risk factors are more susceptible to developing OME including local malignancy, sinonasal disease, loss of ciliary motility, and Eustachian tube dysfunction.^2^, ^3^

MEE in patients with head and neck (HN) cancer is influenced by cancer treatments, particularly radiotherapy and surgical interventions. These treatments often lead to Eustachian tube dysfunction due to swelling, fibrosis, or scarring, which disrupts middle ear ventilation, resulting in fluid accumulation. Radiation therapy has been shown to increase the risk of recurrent MEE and TM perforation due to its damaging effects on surrounding tissues, including the mucosa of the middle ear and Eustachian tube. Studies have indicated that postradiation complications, including chronic Eustachian tube obstruction, contribute significantly to the persistence and recurrence of effusions in this patient population.^4^, ^5^

Treatment options for OME in adults typically begin with conservative approaches, such as intranasal steroids and oral medications. These methods are often preferred due to their noninvasive nature, though their effectiveness can vary significantly among patients.^6^ In cases where conservative treatments are unsuccessful, more invasive procedures like myringotomy with or without tympanostomy tube insertion may be recommended.^7^ Although myringotomy with tube insertion was the most effective treatment for postirradiation OME, it also carried the highest risk of complications.^8^ When comparing myringotomy with ventilation tube insertion versus observation in nasopharyngeal carcinoma (NPC) patients postradiotherapy, myringotomy with ventilation tubes significantly improved hearing outcomes.^9^ Additionally, tube insertion has been shown to yield better outcomes than repeat myringotomies with aspiration in NPC survivors.^10^ To date, however, no published studies have compared the complication rates of myringotomy‐associated procedures between individuals with a history of cancer and those without.

Here, we report a single‐institution retrospective chart review investigating the impact that a history of HN cancer treatment may have on the outcomes that patients with OME experience after a myringotomy procedure with or without tubes.

## Methods

### Data Collection

We performed a retrospective chart review of all adult patients who received a diagnosis of OME at our institution between January 2018 and December 2022 at an academic tertiary care medical center. University of Pennsylvania School of Medicine Institutional Review Board approval was obtained before the collection of data. Clinical variables collected from the electronic medical record include age, gender, race, smoking history, history of HN cancer (cancer type, naso/oropharyngeal reconstruction, radiation dosage, and date of radiation completion), prior history of tympanostomy tubes in childhood and/or adulthood, date of effusion diagnosis, effusion laterality, audiological evaluation results at time of diagnosis and last follow‐up (date of evaluation, air conduction pure‐tone audiometry (PTA), bone conduction PTA, and word recognition score), effusion intervention (tympanostomy tube or myringotomy without a tube), procedure date, date of last follow‐up, and outcome of affected ear per last follow‐up. In cases for which patients were originally treated with a myringotomy without a tube but then subsequently received a tympanostomy tube, the intervention was categorized according to tube placement. When patients required multiple tubes, the number of repeat placements was obtained. Subjects were excluded for prior history of ear surgery ipsilateral to the MEE, including tympanoplasty or mastoidectomy. Audiological evaluations were excluded if the assessment predated the MEE diagnosis by 2 weeks and did not include an audiologist note suggesting symptomatology in the effusion ear. Otherwise, when missing data were encountered, the value for that variable was marked as nonexistent and excluded from the corresponding analysis.

### Definition of the Cancer Cohort

The study population included individuals with a history of HN cancer, as well as those who had received radiation therapy to the HN area during their treatment for non‐HN primary cancers. Further details are provided in Table 1.

**Table 1:** Cancer Subsites Listed From Most Common to Least Common With Clinical Course Data

Cancer subsite	Patients (n)	Radiation treatment (n, %)	Average radiation dose, cGy	Free‐flap reconstruction (n, %)
Oropharynx	20	20 (100)	6459	7 (35)
Oral cavity	17	14 (82)	5843	11 (65)
Nasopharynx	17	17 (100)	6147	2 (12)
Nasal cavity and paranasal sinuses	17	16 (94)	5794	6 (35)
Salivary glands	6	5 (83)	3806	3 (50)
Nonmelanoma cutaneous	5	5 (100)	5533	0 (0)
Non‐HN	4	2 (50)	4500	0 (0)
Larynx	2	2 (100)	NR	1 (50)
Clival/skull base	2	2 (100)	7920	0 (0)
Brain metastases	1	1 (100)	3000	0 (0)
Mandible/temporal bone metastases	1	1 (100)	2000	0 (0)
Parietal bone metastases	1	1 (100)	3010	0 (0)
Skull base	1	1 (100)	5400	0 (0)
Hypopharynx	1	1 (100)	NR	1 (100)
Total	84	78 (93%)	5728	26 (31)

Abbreviations: HN, head and neck; NR, not reported.

### Definition of Adverse Outcomes

In our study, we aimed to define the potential outcomes following surgical intervention for patients with HN cancer, particularly those undergoing radiation therapy, who may experience a variety of ear‐related complications due to treatment side effects and the nature of the disease. We gathered outcomes from the last known status of the patient at their latest follow‐up. We categorized these outcomes into several distinct groups: *persistent perforation*, where a perforation in the TM fails to heal over time, potentially leading to chronic discomfort or hearing loss; *recurrent effusion*, repeat buildup of fluid in the middle ear after we had classified the ear as resolved; *repeat placements* of tympanostomy tubes, required to manage ongoing effusions or prevent recurrence thereof; and *healed or resolved ear*, indicating successful recovery without further intervention. Lastly, we also assessed the probability of experiencing *any adverse outcome*, encompassing persistent perforation, recurrent effusion, or repeat placements, as these events necessitated continued treatment or surveillance.

### Propensity Score Matching

Propensity score techniques were employed to minimize confounding and bias in observational studies by balancing predetermined covariates between treatment and control groups.^11^ We pursued this given the anticipated mismatch between a smaller cancer cohort and a larger noncancer cohort. The control patients were selected based on similar values in the following variables: age, gender, race, smoking history, childhood history of ear tubes, adult history of ear tubes, and audiogram values. The process began with fitting a logistic regression model, where the treatment status was regressed on a set of covariates to calculate the propensity score, representing the probability of receiving the treatment given those covariates. Once these scores were obtained, they were used to match treated and untreated subjects with similar propensity scores or to weight them accordingly. Nearest neighbor matching was used to match treatment and control groups based on propensity scores. A nearest neighbor algorithm, such as the one implemented with the NearestNeighbors class from the sklearn library,^12^ was used to find the closest matches between individuals in the treatment and control groups. The NearestNeighbors model was fitted on the propensity scores of the control group, using a distance metric to determine the closest match for each individual in the treatment group. The BallTree algorithm was employed for this purpose, efficiently handling the computation of nearest neighbors. Once the model was trained, it identified the nearest control group member for each treatment group individual by calculating distances between their propensity scores. All subjects in the treatment and control groups were paired 1:1 with the same or closest propensity score, and those not matched were excluded from statistical analysis run on the propensity score‐matched cohort.

### Statistical Methods

Statistical analysis was conducted using IBM SPSS Statistics (IBM Corp) and Python 3. Postoperative outcomes, including recurrent effusion, persistent perforation, repeat placements, and any adverse outcomes, were reviewed and stratified as binary variables for both the cancer and control groups. Chi‐square (*χ*²) tests were performed to assess differences in categorical variables between the groups, except for variables with counts <5 when the Fisher exact test was used. Continuous variables, such as age and hearing loss, were converted into binary outcomes based on whether the values were above or below the group mean or median.

A multivariable analysis was then conducted using binary logistic regression to adjust for potential confounders and determine the association between various factors and postoperative outcomes. Independent variables in the model included demographic factors, preoperative hearing levels, and cancer subgroup characteristics, which were selected by expert opinion. Missing data were handled by assigning a value of NaN or 0 when appropriate.

Statistical significance was defined as *P* < .05.

## Results

### Overall Demographics

A total of 578 patients met the inclusion criteria for the study, of which 84 had a cancer or radiation history. In terms of intervention type, 512 patients (88.6%) underwent tube placement (either unilateral or bilateral), whereas 66 patients (11.4%) had myringotomy without tube placement. In the overall cohort, the sex distribution was relatively balanced. In terms of racial demographics, the majority of patients were white (65.2%) or black (22.0%) (Table 2).

**Table 2:** Patient Demographics in the Overall Cohort

Patient characteristics	N (%)	Mean (SD)	Range
Total patients	578		
Age at diagnosis, y		59.8 (15.4)	16‐95
Age range (50% of cohort)			51.0‐70.8
Sex			
Male	302 (52.2)		
Female	276 (47.8)		
Race			
White	377 (65.2)		
Black	127 (22.0)		
Asian	28 (4.8)		
Other	22 (3.8)		
Intervention type			
Tube placement only	512 (88.6)		
Myringotomy without tube	66 (11.4)		

Abbreviation: SD, standard deviation.

### HN Conditions

Out of the 84 patients, 78 (92.9%) patients had HN cancer, and the same number received radiation treatment. Furthermore, 26 patients (31.0%) underwent free‐flap reconstruction. On average, cancer patients were older and had a higher proportion of white patients (Table 3). The most commonly affected primary cancer subsites were oropharynx (n = 20), followed by equal numbers of oral cavity (n = 17), nasopharynx (n = 17), and nasal cavity/paranasal sinuses (n = 17) (Table 1).

**Table 3:** Patient Demographics in the Cancer Cohort

Patient characteristics	N (%)	Mean (SD)	Range
Total patients	84		
Age at diagnosis, y		62.6 (10.6)	32‐86
Age range (50% of cohort)			56.8‐68
Sex			
Male	50 (59.5)		
Female	34 (40.5)		
Race			
White	62 (73.8)		
Black	10 (11.9)		
Asian	8 (9.5)		
Other	4 (4.8)		
Intervention type			
Tube placement only	73 (86.9)		
Myringotomy without tube	11 (13.1)		

Abbreviation: SD, standard deviation.

### Analysis of Adverse Outcomes

Within intervention subgroups, persistent effusion was more common in the myringotomy group compared to the tube group (3.9% vs 8.3%, *P* < .05) (Table 4). When stratified by cancer status, tube patients with cancer had significantly higher rates of persistent perforation (16% vs 2%, *P* < .001) and overall adverse outcomes (22% vs 5%, *P* < .001) compared to noncancer patients receiving tubes. Among myringotomy patients, neither persistent perforation (0% vs 4%, *P* = .68) nor overall adverse outcomes (9% vs 16%, *P* = .59) differed significantly between cancer and noncancer patients (Table 5).

**Table 4:** Comparison of Outcomes by Intervention Type in the Overall Cohort

Adverse outcomes	Tube placement (%)	Myringotomy only (%)	Total (%)	*P*‐value
Persistent perforation	22 (4)	2 (3)	24 (4)	.640
Persistent effusion	**20 (4)**	**8 (12)**	**28 (5)**	**<.05**
Any adverse outcome^a^	42 (8)	10 (15)	52 (9)	.098

The bold values indicate the statistical significance *p* < 0.05.

^a^ Any adverse outcome is defined as either persistent perforation or persistent effusion.

**Table 5:** Comparison of Outcomes by Intervention Type in the Cancer Cohort Versus the Noncancer Cohort

	Tube placement			Myringotomy only		
Adverse outcomes	Cancer cohort n = 73 (%)	Control n = 505 (%)	*P*‐value	Cancer cohort n = 11 (%)	Control n = 55 (%)	*P*‐value
Persistent perforation	**12 (16)**	**10 (2)**	**<.001**	0 (0)	2 (4)	.683
Persistent effusion	4 (6)	16 (3)	.333	1 (9)	7 (13)	.763
Any adverse outcome^a^	**16 (22)**	**26 (5)**	**<.001**	1 (9)	9 (16)	.590

The bold values indicate the statistical significance *p* < 0.05.

^a^ Any adverse outcome is defined as either persistent perforation or recurrent effusion.

Persistent perforation was observed in 14% of patients in the cancer subgroup compared to 2% in the control group (*P* < .001). Recurrent effusion occurred in 6% of the cancer or radiation group and 5% of the control group (*P* = .81), showing no significant difference between the groups. Repeat tube placement was required in 35% of the cancer or radiation group versus 42% in the control group (*P* = .24), suggesting no substantial variation in this outcome. Finally, 44% of patients in both groups experienced any adverse outcome (*P* = 1.0). Table 6 highlights the associations between these outcomes and patient characteristics, with persistent perforation being the sole statistically significant difference between the groups.

**Table 6:** Comparison of Cancer Subgroup to Overall Control Group

Adverse outcomes	Cancer or radiation (%)	Control (%)	Total (%)	*P*‐value
Mean repeat tube placements (SD)	4.2 (4.0)	4.3 (4.1)	‐	‐
Persistent perforation	**12 (16)**	**12 (2)**	**24 (4)**	**<.001**
Persistent effusion	5 (6)	23 (5)	28 (5)	.81
Repeat tube placement	29 (35)	208 (42)	237 (41)	.24
Any adverse outcome	37 (44)	218 (44)	270 (47)	1

The bold values indicate the statistical significance *p* < 0.05.

### Propensity Score Matching and Analysis

**Figure 1:**
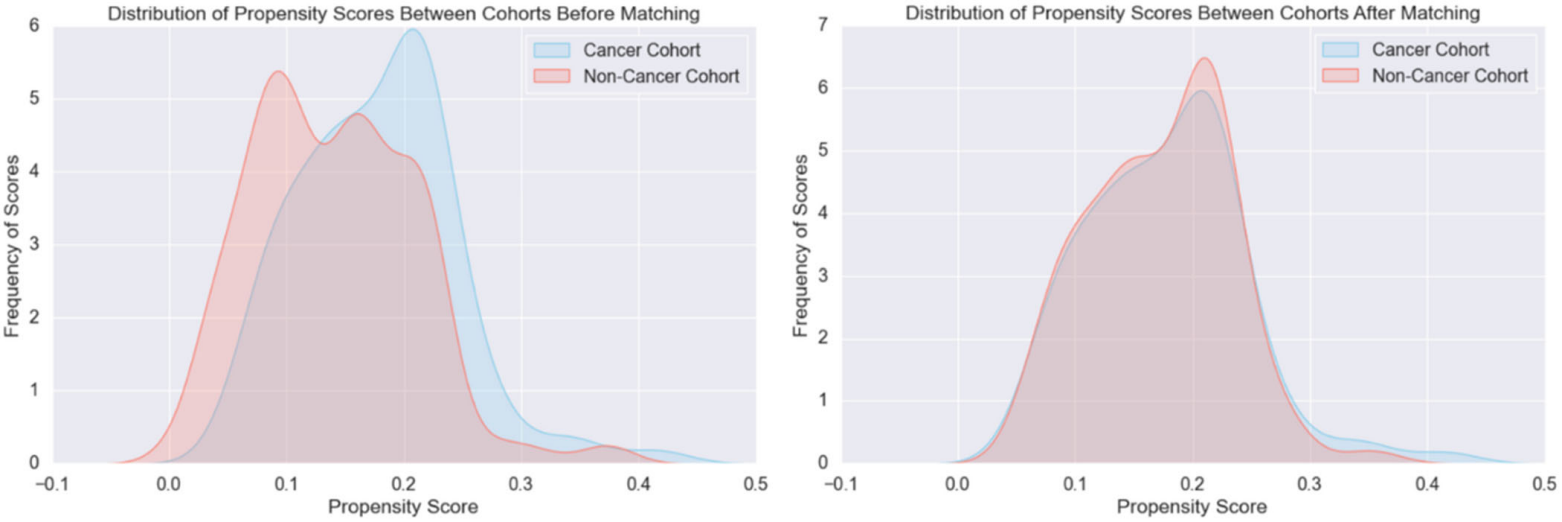
Diagram illustrating the distribution of propensity scores between cohorts before and after matching. Left is prematching; right is postmatching.

Figure 1 shows the distribution of patients before and after propensity score matching. In the propensity score‐matched cohort, *persistent perforation* was significantly higher in the cancer subgroup (14%) compared to the control group (1%) (*P* < .01). However, *recurrent effusion* (6% vs 1%, *P* = .22), *repeat tube placement* (35% vs 46%, *P* = .16), and *any adverse outcome* (44% vs 48%, *P* = .77) showed no statistically significant differences between the groups (Table 7). These findings suggest that cancer or radiation increases the risk of perforation but does not significantly impact other outcomes.

**Table 7:** Comparison of Cancer Subgroup to Propensity Score‐Matched Control Group

Outcomes	Cancer or radiation (%)	Control (%)	Total (%)	*P*‐value
Mean repeat tube placements (SD)	4.2 (4.0)	4.5 (3.4)	‐	‐
Persistent perforation	**12 (14)**	**1 (1)**	**13 (8)**	**<.01**
Persistent effusion	5 (6)	1 (1)	6 (4)	.22
Repeat tube placement	29 (35)	38 (46)	67 (40)	.16
Any adverse outcome	37 (44)	39 (48)	76 (46)	.77

The bold values indicate the statistical significance *p* < 0.05.

### Multivariate Analysis

**Table 8:** Multivariate Analysis of Variables for Each Outcome

	Persistent perforation		Recurrent effusion		Repeat placements		Any adverse outcome	
	OR	*P*‐value	OR	*P*‐value	OR	*P*‐value	OR	*P*‐value
*Female*	1.831	.194	0.601	.242	0.83	.291	0.824	.266
Asian	0.262	.329	0	.998	2.049	.166	1.874	.221
Black	0.542	.515	6.588	.077	1.106	.792	1.489	.289
Hispanic	0	.999	0	.999	0.939	.961	0.764	.834
White	0.474	.369	1.381	.765	1.328	.427	1.338	.404
Former smoker	2.132	.368	0.499	.305	**1.793**	**.038**	1.634	.073
Never smoked	2.701	.210	1.012	.983	1.381	.217	1.522	.097
Current smoker	0	.998	1.615	.521	1.244	.584	1.404	.380
Age over the mean	1.444	.467	1.326	.514	**0.637**	**.019**	0.707	.066
Affected ear air conduction above median	**3.233**	**.041**	1.173	.770	0.932	.769	1.141	.58
Affected ear bone conduction above median	0.510	.292	0.488	.223	0.967	.896	0.788	.336
Affected ear word recognition below median	0.780	.682	0.736	.595	0.857	.507	0.782	.282
Need for free‐flap reconstruction	1.125	.881	1.080E8	.998	1.281	.641	1.725	.284
HN cancer or radiation history	**7.830**	**.007**	0	.998	0.625	.333	0.813	.657
Need for radiation treatment	0.600	.501	0.330	.361	0.820	.686	0.646	.351

The bold values indicate the statistical significance *p* < 0.05.

Abbreviations: HN, head and neck; OR, odds ratio.

**Table 9:** Multivariate Analysis of Variables for Each Outcome Including Cancer Subsites

	Persistent perforation		Recurrent effusion		Repeat placements		Any adverse outcome	
	OR	*P*‐value	OR	*P*‐value	OR	*P*‐value	OR	*P*‐value
*Female*	1.941	.162	0.617	.252	0.820	.263	0.817	.246
Asian	0.664	.764	0	.998	1.749	.285	1.778	.267
Black	0.678	.691	7.535	.060	1.087	.829	1.523	.264
Hispanic	0	.999	0	.999	0.803	.864	0.719	.797
White	0.769	.761	1.475	.720	1.303	.460	1.374	.365
Former smoker	2.722	.238	0.527	.344	**1.832**	**.032**	1.712	.051
Never smoked	2.851	.184	1.077	.896	1.404	.194	1.550	.083
Current smoker	0	.998	1.616	.523	1.286	.528	1.435	.352
Age over the mean	1.268	.642	1.266	.587	**0.633**	**.018**	0.701	.059
Affected ear air conduction above median	2.970	.064	1.185	.763	0.934	.78	1.095	.706
Affected ear bone conduction above median	0.463	.219	0.504	.243	0.961	.875	0.798	.365
Affected ear word recognition below median	0.801	.713	0.747	.620	0.888	.611	0.807	.350
Oral cavity	1.372	.779	3.976	.116	0.743	.578	0.734	.551
Oropharynx	0.986	.990	1.222	.854	0.410	.096	0.520	.179
Nasal cavity and paranasal sinuses	**10.776**	**<.001**	3.778	.118	0.662	.467	1.860	.239
Nasopharynx	2.400	.343	1.043	.971	2.060	.166	1.830	.254

The bold values indicate the statistical significance *p* < 0.05.

Abbreviation: OR, odds ratio.

We examined possible confounders or predictors for adverse events in our cohort (Table 8). For persistent perforation, two variables were statistically significant: higher air‐conducted audiometric thresholds in the affected ear (odds ratio [OR] = 3.23, *P* = .041) and HN cancer or radiation history (OR = 7.83, *P* = .007). Additionally, the nasal cavity and paranasal sinuses subsite was strongly associated with an increased likelihood of persistent perforation (OR = 10.8, *P* < .001) (Table 9). For repeat tube placements, age over the mean was significantly associated with a decreased likelihood of needing repeat procedures (OR = 0.637, *P* = .019), whereas former smoking status had an increased likelihood (OR = 1.79, *P* = .038). In summary, factors such as hearing thresholds, cancer history, and specific cancer subsites like the nasal cavity and paranasal sinuses are significant predictors of ear‐related complications, whereas variables like race and age indicated potential trends but were less definitive.

## Discussion

### Overall Findings and Risk of Persistent TM Perforation

Our analysis revealed that cancer patients face a significantly higher risk of persistent TM perforation following myringotomy with or without tube placement (14% vs 2% overall, *P* < .001; 14% vs 1% after propensity score matching, *P* < .01). After propensity score matching with the noncancer cohort, recurrent MEE did not reach significance (6% vs 1%; *P* = .22).

Prior literature looking at the rates of perforation after myringotomy and tube placement have looked mostly at the type of tube and the duration of retention after placement. One study reported persistent perforations in 3% of cases, with higher rates observed among younger children, those with recurrent purulent otitis media, and those using long‐term Goode T tubes.^13^ Another found that the rate of persistent perforation was significantly higher with long‐term tubes (20%) compared to short‐term tubes (6.6%).^14^ These studies have similar overall rates of persistent perforation as ours, at 4%.

However, prior studies had several key limitations that constrained the scope and generalizability of their findings. Some focused solely on pediatric populations, whereas others focused solely on examining cancer patients without comparing the observed outcomes to a control group of noncancer individuals. In turn, this limits the ability to discern the unique impact of HN malignancy and associated treatments on myringotomy or tube insertion outcomes. Some studies have focused on patients specifically with NPC, overlooking potential differences across cancer types and locations. Certain investigations utilized tympanostomy with aspiration rather than the more commonly employed approach of myringotomy with tube placement, further influencing the reported rates and patterns of TM perforation. Finally, some publications involved highly homogeneous patient populations, restricting the diversity of the sample and the broader applicability of the results. Our present study aims to focus in on a specific intervention—myringotomy with or without tube placement—with clear but more broadly defined inclusion criteria and a control group of patients with no cancer history who underwent the same procedure. Furthermore, we employ propensity score matching to better assess adverse outcome rates after controlling for other demographic features, a statistical strategy that has not yet been employed in the literature on this topic.

### Analysis of Intervention Types

Analysis of intervention‐specific outcomes revealed that although tube placement was generally more effective than myringotomy alone in the overall population, particularly for preventing recurrent effusion, this advantage was substantially modified by cancer status. The presence of HN cancer appeared to reverse this benefit, with cancer patients showing markedly higher rates of complications after tube placement. Although the small number of cancer patients receiving myringotomy alone limits direct comparison between interventions in this population, our findings suggest that the risk‐benefit profile of each intervention varies significantly based on cancer status and should be carefully considered in treatment planning.

### Limited Predictive Role of HN Cancer or Radiation in Multivariate Models

Another notable finding in our study was that although HN cancer and radiation treatment were associated with an increased perforation risk, it was not the only variable that was significant. This suggests that although these factors contribute to the overall clinical picture, other variables, particularly hearing loss and age, may also play a significant contributory role in determining outcomes.

Radiation therapy, a widely used treatment for HN cancers, can have significant adverse effects on the Eustachian tube.^15^, ^16^, ^17^ Studies have reported an incidence of OME ranging from 8% to 29% in these patients undergoing radiotherapy.^18^ One found a correlation between radiation dose to the Eustachian tube isthmus and the development of radiation‐induced OME, with reduced incidence when the dose is kept below 52 Gy for the isthmus and 46 Gy for the middle ear cavity. Another study reported that 22 out of 25 Eustachian tubes functioning normally before radiation therapy shifted to the dysfunction group after treatment, indicating radiation's profound impact on tube function.^19^

When examining adult cancer patients experiencing OME after sinonasal surgery, two key risk factors emerged: posterior surgical resection and postoperative radiotherapy. Patients with posterior resection had a 5.7‐fold increased odds of MEE, whereas those receiving postoperative radiation had an even higher 8.691‐fold risk.^20^ This suggests the mechanical effects of surgery and radiation on the Eustachian tube play a critical role in OME development.

These findings highlight the significant impact of radiation and surgical interventions on the Eustachian tube, leading to increased OME and MEE risks in HN cancer patients. Postradiation OME also remains a common adverse effect in NPC, though clear risk factors were not identified.^21^

Our multivariate model supports a more comprehensive understanding of risk beyond a binary assessment of “cancer vs noncancer,” such that patients with a history of cancer or radiation nonetheless do remain at higher risk for complications that may not be fully captured by standard predictive models. In other words, the development of adverse outcomes in the treatment of OME appears to be a multifactorial process in which a history of cancer is a singular significant factor but not the sole determining one. This multifactorial nature is particularly evident in our finding that higher air conduction thresholds were associated with an increased risk of persistent perforation (OR = 3.23, *P* = .041). This association likely reflects several contributing factors: elevated thresholds may indicate more severe underlying pathology from cancer and radiation treatment affecting the middle ear system; age‐related changes to the TM's healing capacity could play a role, as our cancer cohort was significantly older (62.6 vs 59.3 years, *P* < .05); and these thresholds could indicate more significant Eustachian tube dysfunction, potentially affecting postprocedural healing. Even among patients with a cancer history, only those with malignancies within the nasal cavity and paranasal sinuses exhibited a significantly elevated risk of perforation. For instance, the risk profile for a 30‐year‐old never‐smoker who has undergone multiple ear tube placements differs substantially from that of a 76‐year‐old patient with a low‐risk primary HN cancer site and no radiation history who has never had an ear tube before. This distinction underscores the importance of considering the patient's overall risk profile holistically rather than focusing solely on individual factors such as cancer or hearing loss. Comprehensive assessment of these factors will enable better prediction of outcomes and more personalized approaches to managing OME, particularly in vulnerable populations.

## Limitations and Future Directions

The limitations of our study include retrospective design, single‐institution study design, and relatively small sample size of the cancer cohort. We acknowledge the imbalance between the cancer subgroup and the control, though we also expect that any similar study design would yield a much larger sample of noncancer chronic otitis media than in the cancer cohort. In this context, we promote consideration for the application of propensity score matching to further glean insights into adverse outcomes in mismatched populations of this kind. Only patients who opted to undergo myringotomy and tube were included in this study, limiting the sample size and assessment of the observation cohort. Lastly, decisions regarding otologic management were made at the discretion of the patient and provider rather than following any specific study protocol or division‐wide protocol for management. Future efforts may aim to increase the sample size of the cancer subgroup, including larger, multi‐institutional prospective cohort study or longer period of study.

## Conclusion

The development of adverse outcomes after myringotomy and tube placement is multifactorial. Hearing loss and cancer history are significant predictors of the risk for persistent TM perforation in patients undergoing myringotomy tube placement. Patients with a prior history of HN cancer and radiation to the HN for non‐HN cancers are at higher risk of persistent perforation after myringotomy and tube placement but not at a higher risk for recurrent effusion or repeat tube placements.

## Author Contributions

**Krithika Kuppusamy**, data collection, data analysis, manuscript preparation, manuscript review; **Carly Y. Yang**, data collection, manuscript preparation, manuscript review; **Kevin Wong**, data analysis, manuscript review; **Douglas C. Bigelow**, manuscript review; **Michael J. Ruckenstein**, manuscript review; **Steven J. Eliades**, study design, data analysis, manuscript review; **Jason A. Brant**, data analysis, manuscript review; **Tiffany Hwa**, study design, data analysis, manuscript preparation, manuscript review.

## Disclosures

### Competing interests

The authors declare that there is no conflict of interest.

### Funding source

None.

## References

[1] Rosenfeld RM , Shin JJ , Schwartz SR , et al. Clinical practice guideline: otitis media with effusion (update). Otolaryngol Head Neck Surg. 2016;154(1 suppl):S1‐S41. 10.1177/0194599815623467 26832942

[2] Mills R , Hathorn I . Aetiology and pathology of otitis media with effusion in adult life. J Laryngol Otol. 2016;130(5):418‐424. 10.1017/S0022215116000943 26976514

[3] Kubba H , Pearson JP , Birchall JP . The aetiology of otitis media with effusion: a review. Clin Otolaryngol Allied Sci. 2000;25(3):181‐194. 10.1046/j.1365-2273.2000.00350.x 10944048

[4] Hsin CH , Chen TH , Liang KL , Tseng HC , Liu WS . Postirradiation otitis media with effusion in nasopharyngeal carcinoma patients treated by intensity‐modulated radiotherapy. Laryngoscope. 2013;123(9):2148‐2153. 10.1002/lary.23215 23835775

[5] Na G , Kim KH , Byun HK , Bae SH . Assessment of radiation‐induced otitis media in patients with parotid gland malignancy. Acta Otolaryngol. 2021;141(5):466‐470. 10.1080/00016489.2021.1892184 33719909

[6] Zhong Z , Zhang J , Ren L , Liu Y , Zhen Z , Xiao S . Predictors of conservative treatment outcomes for adult otitis media with effusion. J Int Adv Otol. 2020;16(2):248‐252. 10.5152/iao.2020.8091 32784164 PMC7419099

[7] Steele DW , Adam GP , Di M , Halladay CH , Balk EM , Trikalinos TA . Effectiveness of tympanostomy tubes for otitis media: a meta‐analysis. Pediatrics. 2017;139(6):e20170125. 10.1542/peds.2017-0125 28562283

[8] Xu YD , Ou YK , Zheng YQ , Chen Y , Ji SF . The treatment for postirradiation otitis media with effusion: a study of three methods. Laryngoscope. 2008;118(11):2040‐2043. 10.1097/MLG.0b013e31818208d6 18818551

[9] Charusripan P , Khattiyawittayakun L . The effectiveness of myringotomy and ventilation tube insertion versus observation in post‐radiation otitis media with effusion. Eur Arch Otrhinolaryngol. 2017;274(9):3283‐3290. 10.1007/s00405-017-4617-5 28540514

[10] Chen CY , Hsu WC , Young YH , Hsu MM . Failure of grommet insertion in post‐irradiation otitis media with effusion. Ann Otol Rhinol Laryngol. 2001;110(8):746‐748. 10.1177/000348940111000809 11510732

[11] D'Agostino Jr. RB Propensity score methods for bias reduction in the comparison of a treatment to a non‐randomized control group. Stat Med. 1998;17(19):2265‐2281. 10.1002/(sici)1097-0258(19981015)17:19<2265::aid-sim918>3.0.co;2-b 9802183

[12] 1.1.6. Nearest Neighbors—scikit‐learn 0.16.1 documentation. Sourceforge.net. 2014. Accessed June 15, 2024. https://scikit-learn.sourceforge.net/stable/modules/neighbors.html

[13] Golz A , Netzer A , Joachims HZ , Westerman ST , Gilbert LM . Ventilation tubes and persisting tympanic membrane perforations. Otolaryngol Head Neck Surg. 1999;120(4):524‐527. 10.1177/019459989912000401 10187945

[14] Brown C , Behar P . Factors affecting persistent tympanic membrane perforation after tympanostomy tube removal in children. Int J Pediatr Otorhinolaryngol. 2020;130:109779. 10.1016/j.ijporl.2019.109779 31786523

[15] Wang S , Wang W , Zhang H , Guo M , Hoffman MR , Jiang JJ . Analysis of anatomical factors controlling the morbidity of radiation‐induced otitis media with effusion. Radiother Oncol. 2007;85(3):463‐468. 10.1016/j.radonc.2007.10.007 18006095

[16] Lambert EM , Gunn GB , Gidley PW . Effects of radiation on the temporal bone in patients with head and neck cancer. Head Neck. 2016;38(9):1428‐1435. 10.1002/hed.24267 27453348

[17] Walker GV , Ahmed S , Allen P , et al. Radiation‐induced middle ear and mastoid opacification in skull base tumors treated with radiotherapy. Int J Radiat Oncol Biol Phys. 2011;81(5):e819‐e823. 10.1016/j.ijrobp.2010.11.047 21277110

[18] Christensen JG , Wessel I , Gothelf AB , Homøe P . Otitis media with effusion after radiotherapy of the head and neck: a systematic review. Acta Oncol. 2018;57(8):1011‐1016. 10.1080/0284186X.2018.1468085 29698103

[19] Akazawa K , Doi H , Ohta S , et al. Relationship between Eustachian tube dysfunction and otitis media with effusion in radiotherapy patients. J Laryngol Otol. 2018;132(2):111‐116. 10.1017/S0022215118000014 29343305

[20] Gupta V , Dwivedi G , Sahoo L , et al. Incidence of otitis media with effusion in cases of head and neck malignancies undergoing radiotherapy: a prospective observational study. Indian J Otolaryngol Head Neck Surg. 2019;71(suppl 2):1621‐1625. 10.1007/s12070-019-01698-8 31750227 PMC6841853

[21] Redaelli de Zinis LO , Parrinello G , Schreiber A , Nicolai P . Middle ear effusion in patients with sinonasal cancer treated by surgery with or without radiotherapy. Otolaryngol Head Neck Surg. 2013;148(4):619‐624. 10.1177/0194599812474798 23348873

